# Tissue specific and abiotic stress regulated transcription of histidine kinases in plants is also influenced by diurnal rhythm

**DOI:** 10.3389/fpls.2015.00711

**Published:** 2015-09-11

**Authors:** Anupama Singh, Hemant R. Kushwaha, Praveen Soni, Himanshu Gupta, Sneh L. Singla-Pareek, Ashwani Pareek

**Affiliations:** ^1^School of Computational and Integrative Sciences, Jawaharlal Nehru UniversityNew Delhi, India; ^2^Synthetic Biology and Biofuels Group, International Centre for Genetic Engineering and BiotechnologyNew Delhi, India; ^3^Stress Physiology and Molecular Biology Laboratory, School of Life Sciences, Jawaharlal Nehru UniversityNew Delhi, India; ^4^Plant Molecular Biology Group, International Centre for Genetic Engineering and BiotechnologyNew Delhi, India

**Keywords:** abiotic stress, *Arabidopsis*, histidine kinase, histidine phosphotransfer protein, response regulator, rice, two-component system

## Abstract

Two-component system (TCS) is one of the key signal sensing machinery which enables species to sense environmental stimuli. It essentially comprises of three major components, sensory histidine kinase proteins (HKs), histidine phosphotransfer proteins (Hpts), and response regulator proteins (RRs). The members of the TCS family have already been identified in *Arabidopsis* and rice but the knowledge about their functional indulgence during various abiotic stress conditions remains meager. Current study is an attempt to carry out comprehensive analysis of the expression of TCS members in response to various abiotic stress conditions and in various plant tissues in *Arabidopsis* and rice using MPSS and publicly available microarray data. The analysis suggests that despite having almost similar number of genes, rice expresses higher number of TCS members during various abiotic stress conditions than *Arabidopsis*. We found that the TCS machinery is regulated by not only various abiotic stresses, but also by the tissue specificity. Analysis of expression of some representative members of TCS gene family showed their regulation by the diurnal cycle in rice seedlings, thus bringing-in another level of their transcriptional control. Thus, we report a highly complex and tight regulatory network of TCS members, as influenced by the tissue, abiotic stress signal, and diurnal rhythm. The insights on the comparative expression analysis presented in this study may provide crucial leads toward dissection of diverse role(s) of the various TCS family members in *Arabidopsis* and rice.

## Introduction

Growth potential of the plants are severely affected under various abiotic stress conditions especially salinity and drought. Since plants are rooted to a place, they have to make adjustments in their genetic and metabolic machinery in order to survive under abiotic stress conditions. Under stress conditions, plants use specific signaling machineries to relay the stress signals in order to “switch on” the adaptive responses which assist plants in developing tolerance toward abiotic stress. Some of the signaling machineries are conserved across various genera. One such signaling machinery is the two-component system (TCS) or His-to-Asp phosphorelay which is well-known and conserved machinery for signal transduction in the cells (Mochida et al., 2010; Nongpiur et al., 2012). Apart from stress signaling, TCS has been one of the key regulators for many biological processes such as cell division, cell growth and proliferation, and responses to growth regulators in both prokaryotic and eukaryotic cells (Stock et al., 2000; Hwang et al., 2002; Mizuno, 2005; Pareek et al., 2006; Schaller et al., 2008; Pils and Heyl, 2009).

**Figure 1:**
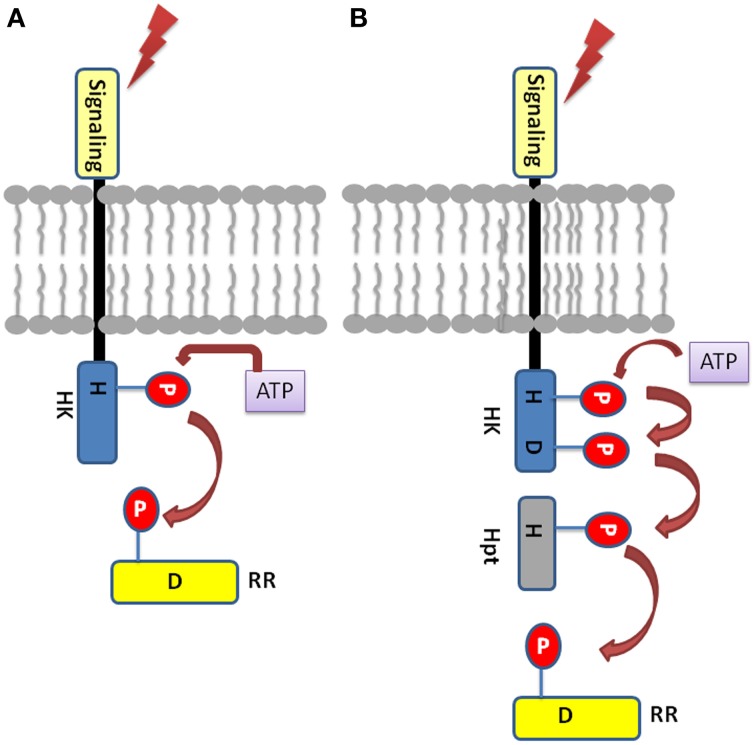
**Basic Two-component system architecture (A) A simple TCS member: The sensory domain of histidine kinase sense the extracellular signal which leads to the phosphorylation of the conserved His in its transmitter domain**. Further, the conserved Asp in the receiver domain of the RR is autophosphorylated resulting in signal output. **(B)** Hybrid-type TCS member: The conserved His and Asp are present in the same protein which is membrane bound. The Hpt protein acts as mediator for the transfer of the phosphoryl group between the HK and the RR.

The TCS signaling system essentially comprise of sensory histidine kinases (HKs) and their cognate response regulators (RRs) substrates, which have been reported in almost all the sequenced bacterial genomes except for mycoplasma (Mascher et al., 2006; Laub and Goulian, 2007). In a simple prototypical TCS regulatory system, HK protein senses the environmental signals, autophosphorylates a histidine residue (H), and signals to its corresponding cytosolic RR protein by transferring the phosphate to an aspartate residue (D) (Figure 1). Phosphorylated RR further mediates the downstream signaling (Urao et al., 2000). Some of the bacteria, yeast, slime molds and plants possess a more complex form of TCS or His-to-Asp phosphorelay system. This is due to the presence of “hybrid” type of kinases which possess both His-kinase (HK) domain and a receiver domain (RD) in one protein. Another protein namely, His-containing phosphotransfer (Hpt) protein is involved which acts as a signaling module connecting to the final RRs (Schaller et al., 2008). Hpts allows species to have multistep phosphorelays which has a major advantage of having multiple regulatory checkpoints for signal crosstalk or negative regulation by specific phosphatases (Urao et al., 2000).

Structurally, Histidine kinase (HK) protein is a dimeric protein and is regulated by receptor-ligand interactions (Grebe and Stock, 1999; Koretke et al., 2000). The HK proteins consist of highly conserved domains, the dimerization and histidine phosphotransfer domain (DHp), which contains the conserved histidine, and the catalytic and ATP binding (CA) domain (Cheung and Hendrickson, 2010). Apart from other functional domains, the HK protein has a sensory domains like HAMP (Histidine Kinases, adenyl cyclases, methyl accepting proteins, and phosphatases), GAF (cGMP-specific phosphodiesterases, adenyl cyclases and FhlA), PAS (Per Arnt Sim), and phytochrome domains for sensing wide range of environmental cues. Response regulators share a common, well conserved receiver domain RD that catalyzes phosphotransfer from its cognate HK (Capra and Laub, 2012). The differential gene expression is the consequence of protein-protein interaction or protein-DNA interaction which is mediated by the C-terminal effector (or output) domain of the RR thus giving rise to the appropriate cellular response (Mascher et al., 2006). In hybrid type kinases, phosphate is first transferred from the histidine residue in the transmitter to the aspartate residue of the attached RD, then to a histidine residue on a histidine phosphotransfer domain (Figure 1). Finally, the phosphate is relayed from the Hpt domain to the RD of a down-stream response regulator protein (RRs), which results in the output response. Based on highly conserved residues which HK proteins possess conserved sequence fingerprints, namely H, N, D, F, and G-boxes can be identified. The H-box bears the histidine that get phosphorylated while the N, D, F, and G-boxes are located at the ATP binding site (Kofoid and Parkinson, 1988; Stock et al., 1988, 1995).

Several plant species, including model plant *Arabidopsis*, are known to possess TCS signaling machinery (Hwang and Sheen, 2001; Grefen and Harter, 2004). Crucial processes such as cytokinin signaling, ethylene signaling, and light perception involves members of the TCS (Hwang et al., 2002). The presence of TCS system in eukaryotes was anticipated in *Arabidopsis* with the characterization of ethylene receptor ETR1 (Chang et al., 1993), photoreceptors (Schneider-Poetsch, 1992; Li et al., 2011) and yeast osmosensor SLN1 (Ota and Varshavsky, 1993) which was earlier considered to be restricted only to prokaryotes. The characterization of multi-step TCS machinery in *Arabidopsis* as the key element of plant cytokinin signaling revealed TCS machinery in plants (To and Kieber, 2008). In *Arabidopsis*, AtHK1 of the TCS family is indicated to be involved in the osmosensing mechanism (Urao et al., 1999). Earlier, we have performed whole genome analysis of the TCS members in rice in comparison to *Arabidopsis* (Pareek et al., 2006). Further, current advances have shown role of TCS machinery in various environmental stresses such as drought, cold, osmotic stress and abscisic acid (ABA) (Tran et al., 2010; Ha et al., 2011; Hwang et al., 2012).

The current investigation presents the comprehensive expression analysis of various members of TCS in *Arabidopsis thaliana* and *Oryza sativa* using massively parallel signature sequencing (MPSS) and publicly available microarray data under various abiotic stress conditions. The analysis would be able to enhance our understanding about the role of TCS members in the two plant species.

## Materials and methods

### Search of TCS members in *Oryza sativa and Arabidopsis*

Earlier, all the members of TCS signaling machinery were identified and characterized in rice (TIGR rice database version 4.0) and were compared to the TCS members present in *Arabidopsis* (Hwang and Sheen, 2001; Grefen and Harter, 2004). The TCS signaling members were retrieved for *Arabidopsis* and rice as done earlier (Pareek et al., 2006) using TIGR rice database version 7.0 and TAIR version 10 for *Arabidopsis*, in order to rule out any new member or deleted member protein from the updated genome database versions.

### Analysis of MPSS database for expression profiles

With the representation of more number of signature libraries in the MPSS database (Brenner et al., 2000), we have extracted expression evidence from the most recent MPSS tags for both *Arabidopsis* and *Oryza* gene models (Database release 2008). With high specificity, the signature sequence uniquely represents a gene and shows perfect match (100% identity over 100% length of the tag). The expression of the gene is estimated by the normalized abundance (tags per million, tpm) of specific signatures in a given library. Class 1, 2, 5, and 7 were used for sense coding sets while Class 3 and 6 were used for antisense coding sets. For both the genomes, 20-nt tags were used for determining the tissue specific expression of TCS members.

In *Arabidopsis*, the tissue specific signature libraries considered for analysis are as follows: for Callus—CAF, CAS; for inflorescence—INF, AP1, AP3, AGM, INS, SAP; for leaves—LEF, LES, S04, S52; for roots—ROF, ROS; for Silique—SIF, SIS; for seeds—GSE. These libraries were earlier considered for analysis of CBS family protein in *Arabidopsis* (Kushwaha et al., 2009).

In rice, the tissue specific signature libraries considered for analysis are as follows: for leaves—I9LA, I9LB, I9LC, I9LD, FLA, FLB, FLC, FLD, NDL, NCL, NLA, NLB, NLC, NLD, NYL, NSL, PLA, PLW, PLC; for meristem—NME, I9ME, FME; for roots—I9RO, I9RR, FRO, FRR, NYR, NRA, NRB, NDR, NCR, NSR; for callus—NCA; for panicle—NIP; for stigma—NOS; for pollen—NPO; for stem—NST; for seeds—NGD, NGS, PSC, PSI, PSL, PSN, PSY. These libraries were earlier considered for analysis of CBS family protein in rice (Kushwaha et al., 2009). The quantitative values obtained for respective TCS genes were used for making the heatmap using open source R software.

### Expression analysis using microarrays

In order to analyze the gene expression for various abiotic stress conditions the latest microarray data for cold, UV, wound, heat, genotoxic, drought, osmotic, salt, and oxidative stress were retrieved from the *Arabidopsis* Information Resource (Lamesch et al., 2012). The tissue (root and shoot) specific datasets were obtained for different time sets namely 30 min, 1 h, 3 h, 6 h, 12 h and 24 h of various abiotic stresses and analyzed, as performed earlier (Kushwaha et al., 2009). The pre-normalized data thus obtained, was used for analysis of fold change expression in *Arabidopsis*. The expression datasets for the rice were obtained from NCBI-GEO database (Supplementary Table 1). The microarray expression data was downloaded using Bioconductor package. The array quality of the experiment was assessed by MA and RNA degradation plot for individual arrays. The individual GEO raw data sets were normalized using RMA method. The normalized datasets were integrated and differentially expressed genes were identified using RankProd package in Bioconductor (Hong et al., 2006). The expression matrix thus obtained, was used to extract expression values for TCS members. Fold increase in transcript abundance under stress conditions were calculated with respect to their respective controls. The expression with respect to the control was calculated using in-house PERL programs. The hierarchical clustering analysis and the heatmaps were made using R software.

### Plant material and growth conditions

Seeds of *Oryza sativa* L. cv “IR-64” were washed with deionized water and allowed to germinate in half Yoshida medium (Yoshida et al., 1972) under hydroponic system with continuous air bubbling for 48 h in dark and then transferred to light for further growth for 14 days under control conditions (28 ± 2°C, 12 h light and 12 h dark cycle) in plant growth chamber, having 70% relative humidity. To find out the rhythmic expression of TCS genes in rice, shoot samples were harvested for 2 days at an interval of 3 h starting from the dawn of 15th day from rice seedlings grown under 12 h light/12 h dark cycle. After harvesting, each sample was immediately frozen in liquid nitrogen and stored at −80°C till further use.

### Isolation of total RNA and cDNA synthesis

Total RNA was isolated from the harvested plant samples using RaFlex™ solution I and solution II (GeNei, India) as per the manufacturer's protocol. Two hundred milligram of each sample was used for RNA extraction. The quantity and quality of RNA was estimated by determining absorbance at a wavelength of 260 nm. Concentration of RNA was calculated using OD_260_ nm formula (OD_260_ = 1, corresponds to 40 μg/ml of RNA). Quality of RNA was checked by A_260_/A_280_ ratio. Five microgram of total RNA of each sample was checked by electrophoresis. EtBr stained formaldehyde agarose gel showed the presence of two distinct bands of 28S rRNA and 18S rRNA in each sample of total RNA. It confirmed the integrity of RNA of each sample which was then used for subsequent cDNA synthesis. First strand cDNA was synthesized using first strand cDNA synthesis kit (Fermentas). Total RNA was treated with DNase to remove genomic DNA contamination. For DNase treatment, 5 μg RNA was incubated with 1 unit of DNase in 1X buffer for 30 min at 37°C. The DNase was then denatured by heating at 75°C for 5 min. Before heating 1 μl of 25 mM EDTA, which works as a chelating agent, was added to the reaction mixture to prevent RNA break down. After this treatment, RNA samples were used for first strand cDNA synthesis. The primers of TCS members were designed using Primer 3 express (Applied biosystem, USA) and NCBI primer BLAST software. The sequences for these primers are listed in Supplementary Table 2. All Primers were specific to the unique regions in the 3′-UTR of their respective genes. These primers were rechecked for their uniqueness via primer BLAST at NCBI database. For primer designing the transcript nucleotide sequences were downloaded from TIGR rice database. Designed primers were ordered to Sigma-Aldrich, India for synthesis.

### Quantitative RT-PCR

The rice translation elongation factor 1α (eEF-1α) gene was taken as the reference gene for the analysis. The quantitative RT-PCR reaction mixture contained 5 μl of 10 fold diluted cDNA, 10 μl of 2X SYBR Green PCR Master Mix (Applied Biosystems, USA), and 100 nM of each gene-specific primers in a final volume of 20 μl. No template controls (NTCs) were also taken for each primer pair. The real-time PCR reactions were performed employing ABI Prism 7500 Sequence Detection System and software (PE Applied Biosystems, USA). All the reactions of quantitative real-time PCR were performed under following conditions: 10 min at 95°C, and 40 cycles of denaturation at 95°C for 25 s, annealing and extension at 59°C for 1 min in 48-well optical reaction plates (Applied Biosystems, USA). The specificity of amplification was tested by dissociation curve analysis. The experiment was repeated with two biological replicates, each of them having three technical replicates. Data analysis was performed using ddCT method (Livak and Schmittgen, 2001).

## Results

The analysis of TCS members has been carried out using latest version of genomes of *Arabidopsis thaliana* (TAIR ver. 10) and *Oryza sativa* (TIGR ver. 7) using pfam profiles (Pareek et al., 2006). In comparison to the earlier report (Pareek et al., 2006), some new members have been found in both *Arabidopsis* and rice, in the current analysis (Supplementary Tables 3A–F). Earlier analysis suggested 54 genes coding for 63 proteins in *Arabidopsis* while current analysis found 54 genes coding for 73 proteins. Similarly in *O. sativa*, 51 genes encoding 73 putative proteins was reported earlier but the current analysis showed 52 genes coding for 81 proteins (Table 1). The new members added to the list of histidine kinase have been named as histidine kinase like (*OsHKL1*) gene because of the presence of only histidine kinase domain in the protein sequence. The increase in the number of protein products has been attributed to the presence of more alternative spliced products. In order to avoid any ambiguity in nomenclature, the previous nomenclature has been retained as reported in Pareek et al. (2006).

**Table 1:** **Comparison of TCS architecture in ***Arabidopsis*** and ***O. sativa*****.

**Gene Family**	***Arabidopsis thaliana***		***Oryza sativa***	
	**No. of genes**	**No. of proteins**	**No. of genes**	**No. of proteins**
TCS	54	73	52	81
HK	15	19	13	22
Hpt	6	9	5	7
RR	33	45	34	52

### Analysis of expression profiles for TCS members in *Arabidopsis* and rice using MPSS database

Sensitive measures of expression of all genes in the genome can be assessed using MPSS (Brenner et al., 2000). MPSS has been used for the analysis of genome-level expression analysis in various plant systems including rice, *Arabidopsis* and grapes (Meyers et al., 2004). To extract information about the relative abundance of transcripts of TCS members in various tissues/organs of *Arabidopsis* and rice, analysis was carried out using MPSS database (http://mpss.udel.edu). Analysis of TCS-specific mRNA tags (measured as transcript per million; TPM) in various libraries showed considerable variability in their abundance in various tissues.

**Figure 2:**
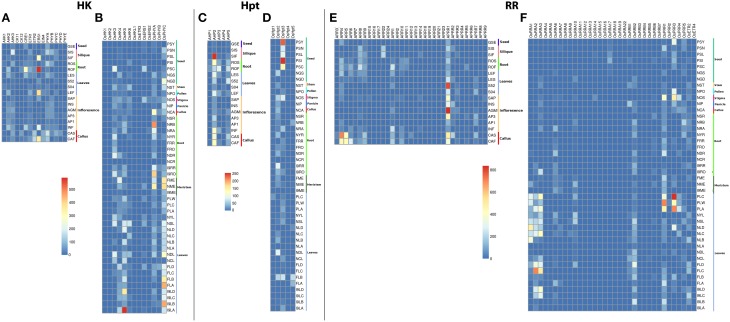
**Heatmap depicting the tissue-specific transcript accumulation for Histidine Kinases (HKs) for (A) ***Arabidopsis*** and (B) rice, Histidine phosphotransfer proteins (Hpts) for (C) ***Arabidopsis*** and (D) rice, Response regulators (RRs) for (E) ***Arabidopsis*** and (F) rice**. These heatmaps are based on the MPSS database. The heatmap was made using open source R software.

Among the HKs, *ERS1* showed considerably large accumulation of transcripts in untreated 21-days roots while it showed moderate rise in transcripts in 28–48 h post fertilized silique, untreated 21-day leaf and actively growing callus in *Arabidopsis* (Figures 2A,B). Another HK, *CRE1* showed moderate accumulation of transcripts in untreated 21-days roots. Low transcripts accumulation was observed in *AHK2, AHK3, ETR1, PHYA*, and *PHYB* in *Arabidopsis*. On the other hand, *OsHK5* showed considerably high accumulation of transcripts in leaves. The transcripts accumulation was also observed in leaves, 60 days mature leaf for *OsPHYc* in rice. The accumulation of transcripts was also observed in 60 days mature roots for *OsPHYA* gene. Among the Hpts in *Arabidopsis, AHP2* showed accumulation of transcripts in 24–48 h post fertilized silique while it shows moderate accumulation in callus. On the contrary, *OsHpt3* in rice showed high accumulation in developing seeds (Figures 2C,D). For the RRs, in *Arabidopsis, APRR1* showed high accumulation of transcripts in agamous inflorescence and leaves while *ARR4* showed transcript abundance in callus. In rice, *OsPRR3* and *OsPRR1* showed accumulation of transcripts in beet armyworm damaged; water weevil damaged and mechanically damaged leaves. Another RR, *OsRRA2* and *OsRRA3* showed transcripts accumulation in 60 days mature leaves (Figures 2E,F). These observations suggest that the TCS-related transcriptome of *Arabidopsis* and rice is complex, showing a tissue-specific differential regulation.

### Analysis of expression profiles for TCS members in *Arabidopsis* and rice using microarray

For analysis of expression of the TCS members in *Arabidopsis*, we used data available on the TAIR database consisting time series analysis performed in root and shoot tissues under various abiotic stress conditions while for rice, the expression was analyzed using various abiotic stress experiment data available at NCBI-GEO database.

#### Histidine kinase proteins (HKs)

**Figure 3:**
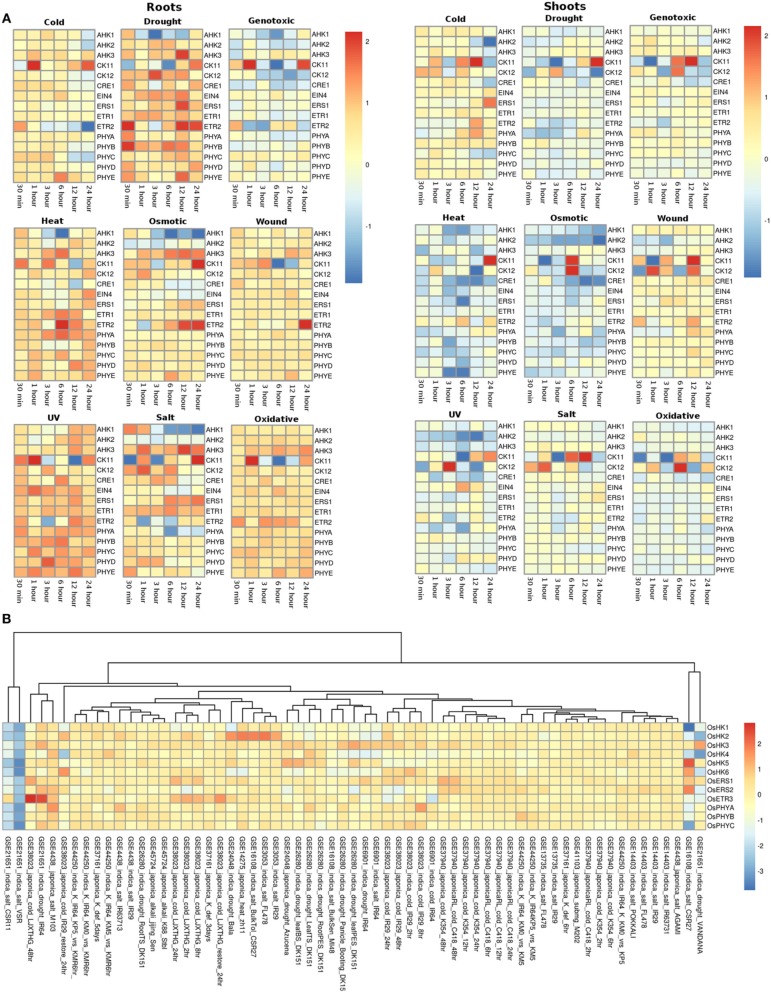
**Heatmap depicting the stress-induced expression of Histidine Kinase (HKs) genes from root and shoot tissues obtained using microarray data for ***Arabidopsis*** (A) and ***O***. ***sativa*** (B)**. The heatmap was made using open source R software.

The expression profile of the histidine kinase genes in root tissues of *Arabidopsis* under cold conditions showed downregulation of *AHK1* and *AHK2* genes at 24 h of stress, while at the other time spans, its expression remained unchanged. On the other hand, in shoots, *AHK1* maintained a basal level of expression in all the cold stress time points but the transcripts for *AHK2* showed two fold downregulation at 12 and 24 h of cold stress conditions (Figure 3A). Another cytokinin signaling gene, *CK11* showed changing expression during all time-series. The gene was observed to be downregulated at 30 min of cold stress and two fold upregulated at 1 h of stress. The expression of this gene gets normalized only to get further upregulated by over two folds at 24 h of cold stress. Further, in shoots, *CK11* gene showed similar behavior where the basal level of expression is maintained at 30 min of cold stress, which gets upregulated upto two folds in 1 h of stress. The expression further goes down two folds at 3 h of stress and again gets two fold upregulated at 6 h and 12 h of stress. Finally, it again shows downregulation at 24 h of cold stress (Figure 3A). *CK11* and *AHK1* have been found to play major role in the cytokinin signaling and osmosensing process respectively in *Arabidopsis* (Urao et al., 2000). In roots, ethylene receptor, *ETR2* showed downregulated response as the time span of the cold stress increases from 30 min to 24 h while, in shoots, it shows an upregulated response till 12 h of cold stress and finally maintains a basal expression at 24 h of cold stress. In shoots, another member of TCS, namely *AHK3*, showed downregulated response at 24 h of cold stress and *CK12* showed upregulation in 30 min, 1 h and 6 h of cold stress while it gets downregulated in 3 h of cold stress. *ETR2* also showed upregulation in response to 12 h of cold stress. Among the photoreceptors, *PHYC* showed downregulation in 12 and 24 h of cold stress in both root and shoot tissues. In roots, photoreceptor *PHYE* showed upregulation in 6 h of cold stress, while it maintained basal level expression in the shoot tissues.

Under drought conditions, in the root tissues, all the histidine kinase genes in *Arabidopsis* were found to be upregulated. *AHK1* was found to be upregulated in 30 min of drought stress followed by its downregulation in 1, 3, 6, and 12 h of drought stress, after which it again got upregulated by two folds in 24 h. Ethylene receptor *ETR2*, showed two fold upregulation at 30 min, 12 h and 24 h of drought stress, photoreceptor *PHYB* showed upregulated response at 30 min of drought stress. On the other hand, in shoots of *Arabidopsis*, most of histidine kinase genes showed downregulation in response to drought stress. Only *CK11* showed three fold upregulation in 12 h of drought stress and then got downregulated in continuation of drought stress for 24 h.

Under the genotoxic stress conditions, in root tissue, genes namely, *AHK1, AHK2, CK12, CRE1*, and *ETR2* showed downregulated response. Rest all the members of the HK family showed minimal level of expression at all the time points in both root and shoot tissues. Only *CK11* showed three fold upregulation in 1 h of genotoxic stress and got downregulated only to get upregulated again at 24 h of stress. In shoot tissues, *CK11* gene showed upregulation at 6 and 12 h of genotoxic stress. Another gene, *CK12* showed 1.5-fold upregulation at 6 h of stress.

Similar response of the members of the HK family was found in response to heat stress. In root tissues, all genes of HK family maintained minimal to high expression at all time points of the stress. Specifically, gene *ETR2* showed three fold upregulation at 6 h of stress while *AHK1* showed downregulation at 3 h and 6 h of heat stress condition. On the other hand, in shoot tissues, the entire HK family members showed exactly the reverse of the expression as it showed in root tissues, that is, mainly down expression for most of the members. Only, *CK11* showed two fold upregulated expression at 24 h of heat stress.

Under osmotic stress, in root tissues, *CK11* and *ETR2* showed three fold upregulation during 24 h of osmotic stress while *AHK1* showed downregulated expression during the 3, 6, 12, and 24 h of osmotic stress. *CK11* and *CK12* showed upregulation during 6 h of osmotic stress in the shoot tissues. All the other members showed similar level of expression as in the heat stress, that is, downregulation for most of the members. Under the wounding stress, *CK11* and *CK12* showed upregulated response in 1 h and 12 h of stress in the shoot tissues while in root tissues, only *ETR2* showed two fold upregulation in the expression. Rest of the members of the HK family maintained unchanged expression levels at all time points in both root and shoot tissues.

Similar to heat and osmotic stress conditions, HK members showed minimal to high expression under UV stress in the root tissues while in shoot tissues, *CK12* showed three fold upregulation in 3 h of stress. In roots, *AHK1* showed downregulation after 6 h of UV stress condition. Further, under salt stress conditions, all the HK members were found to be upregulated except *AHK1* and photoreceptor, *PHYA* in the root tissue while in shoot tissues, the expression of HK members remained unchanged with respect to the control conditions. *CK11* and *CK12* showed high expression in the salt stress condition in shoot tissues. Similar expression profile was observed in oxidative stress conditions in both root and shoot tissues. Overall, the HK family members in *Arabidopsis* appear to be more active in the root tissues than in the shoot tissues in all the stress conditions.

In rice (in all the experiments and genotypes of rice) histidine kinases (HKs) showed similar behavior of basal expression under cold conditions. Only in GEO dataset, GSE38023, ethylene receptors *OsERS1, OsERS2*, and *OsETR3* showed one to two fold upregulation at various time points (Figure 3B). Under drought conditions, in indica genotype of rice Vandana (GSE21651), *OsHK3* showed 1.5-fold upregulation while *OsHK2* and *OsHK3* showed similar fold downregulation in the expression. In IR64 genotype (GSE21651), *OsETR3* showed two fold upregulation under drought stress. All the other members of histidine kinase gene family showed basal expression levels in all other experiments. In salt stress conditions, all the members of the HK gene family maintained basal expression in all the experimental conditions. In two of the rice genotypes, VSR and CSR11 all the HK members were found to be downregulated under salt stress conditions.

#### Histidine phosphotransfer proteins (Hpts)

**Figure 4:**
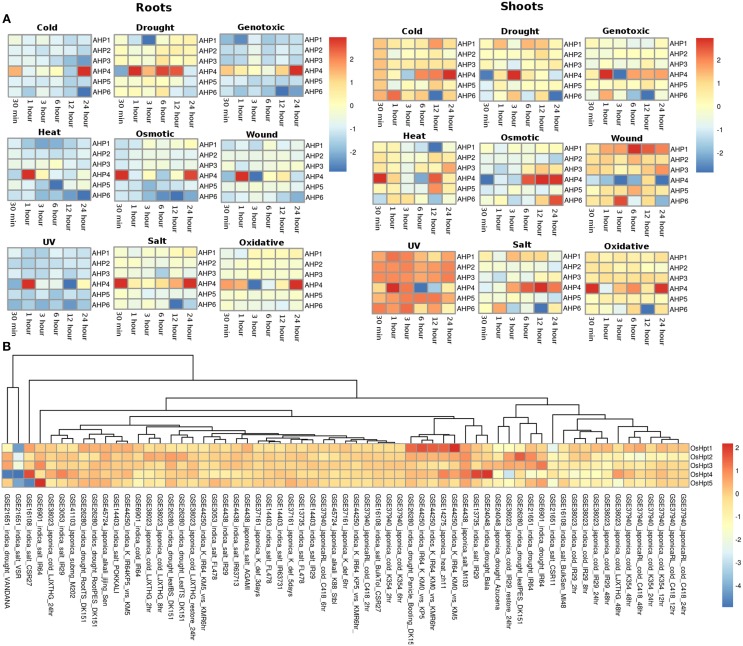
**Heatmap depicting the stress-induced expression of Histidine phosphotransfer protein (Hpts) genes from root and shoot obtained using microarray data for ***Arabidopsis*** (A) and ***O. sativa*** (B)**. The heatmap was made using open source R software.

The expression profiles of the Histidine phosphotransfer (Hpt) genes in *Arabidopsis* in root and shoot tissues appear to show reverse behavior as compared to the HK family members under all the stress conditions. Overall, Hpt family members appear to show higher expression in shoots than in roots under all the considered abiotic stress conditions (Figure 4A). Under osmotic stress conditions, in the root tissues, all the Hpt members appear to be downregulated except for *AHP4* which showed three fold upregulation. Under osmotic stress, Hpt members namely *AHP1, AHP2*, and *AHP3* showed downregulation at 30 min and 1 h of stress but they maintained a basic level of expression at other time points in the root tissues. While in shoot tissues, all the genes were observed to be expressed at all the time points especially, *AHP4* and *AHP6* showed high (two or three fold) upregulation under osmotic stress. Under drought stress conditions, all the Hpt members showed similar expression in root as well as shoot tissues. Again, *AHP4* showed high upregulation at 1, 6, and 12 h of drought stress in roots and at 3 h in shoots. Under genotoxic conditions, all the members, with an exception of *AHP4* showed downregulation in the root tissues. In shoot tissues, members of the Hpt family showed unchanged minimal expression with respect to the control conditions. Similar to cold stress, all the members showed downregulation all time points except for *AHP4* which showed three fold upregulation during 1 h of stress in the root tissues. However, in the shoot tissues, all the Hpt members were observed to be upregulated except for *AHP6* which showed two fold downregulation in 12 h cold stress (Figure 4A). On the other hand, all the members of Hpt family maintained minimal expression except for *AHP4* which showed three fold upregulation at 30 min of heat stress. Under wounding stress, in roots, all the members were downregulated while in shoot tissues all the Hpt genes were upregulated. *AHP4* was found to be downregulated under the wounding stress at all the time points in the shoot tissues. *AHP1* showed higher expression under the wounding stress in the shoot tissues. Under the UV stress, Hpt members showed exactly reverse expression behavior in the root and shoot tissues with the expression of *AHP4* showing periodic pattern in the expression. Under salt and oxidative stress conditions, the expression levels remained same in both root and shoot tissues showing that the salt stress nearly leads to the oxidative stress as well. Overall, it appears that the *AHP4* gene of the Hpt family is most active members among all, showing expression in all the considered abiotic stress conditions.

In rice, the members of the Hpt family showed expression in all the considered experimental conditions in all the considered genotype suggesting their potential importance in the rice plant (Figure 4B). *OsHpt4* showed three fold expression in drought and salt stress conditions in Bala (GSE24048) and IR29 (GSE13735) genotypes of rice respectively. *OsHpt4* showed three folds expression under salt stress in the CSR27 genotype of rice (GSE16108) while *OsHpt5* showed three folds expression under similar conditions in IR64 genotype of rice (GSE6901). In other genotypes namely, VSR and Vandana, *OsHpt4* showed two fold downregulation under salt and drought stress (GSE21651).

#### Response regulator proteins (RRs)

**Figure 5:**
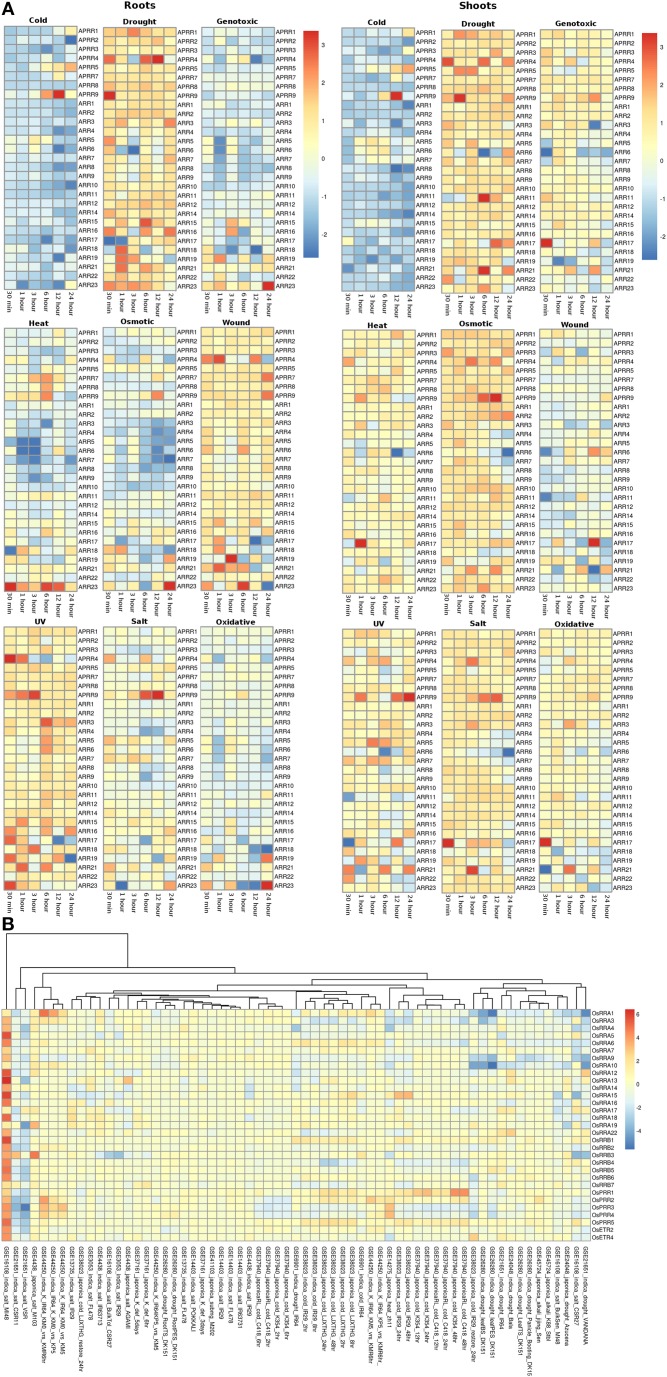
**Heatmap depicting the stress-induced expression of Response regulator (RRs) genes from root and shoot obtained using microarray data for ***Arabidopsis*** (A) and ***O***. ***sativa*** (B)**. The heatmap was made using open source R software.

The RR gene family members in root and shoot tissues of *Arabidopsis* showed similar pattern of expression under various abiotic stresses considered in this investigation. Under the cold stress, *APRR9* showed three fold upregulation in 12 h of stress in both root and shoot tissues. Most of the members of the RR family were found to be downregulated under cold stress conditions in both root and shoot tissues (Figure 5A). Under drought conditions, most genes of the RR family maintained minimal expression in both root and shoot conditions. Genes namely *APRR4* (*Arabidopsis* pseudo response regulator), *APRR9* and *ARR15* were upregulated in the root tissues while *APRR4, APRR9, ARR11*, and *ARR21* were upregulated in the shoot tissues under cold stress conditions at various time points. In genotoxic stress conditions, most gene members of the family were downregulated in the root tissues, while in shoots, most of the genes maintained unaltered expression at various time points. Similar expression pattern was observed in response to heat and osmotic stress. Only *ARR2*3 in the root tissues was found to be upregulated while in shoots, it maintained unchanged expression with respect to control conditions. Under osmotic stress, *APRR9* in the shoot tissues got upregulated in 12 h of stress. Under wounding stress, most of the members maintained unaltered expression at various time points in the root tissues while in shoot tissues, RR gene family members showed mixed response ranging from downregulation to unaltered expression. In response to UV stress, RR family members maintained unchanged expression with respect to the control conditions with only *APRR4, APRR9*, and *ARR3* showing upregulated expression in the root tissues while in shoot, only *APRR9* showed three fold upregulation. In salt stress conditions, RR gene family members in shoot tissues were found to have marginally higher expression levels than in the root tissues. Pseudo response regulator, *APRR9* showed two fold and three fold high expression in the shoot and root tissues respectively. Similar pattern of expression was found in the oxidative stress of both root and shoot tissues of *Arabidopsis*.

In rice, similar to *Arabidopsis*, all the members of RR gene family showed unaltered expression under all the abiotic stress conditions in all the genotypes. *OsRRA1* showed downregulation in the indica genotype under salt and drought conditions (Figure 5B). Some genes of the RR gene family showed downregulation in the indica genotype in VSR and CSR11 (GSE21651) variety of rice. Another variety of the indica genotype, *MI48* showed two to three fold upregulation of various gene members of the RR gene family (GSE16108).

### qRT-PCR-based analysis of TCS members toward diurnal rhythm in rice

**Figure 6:**
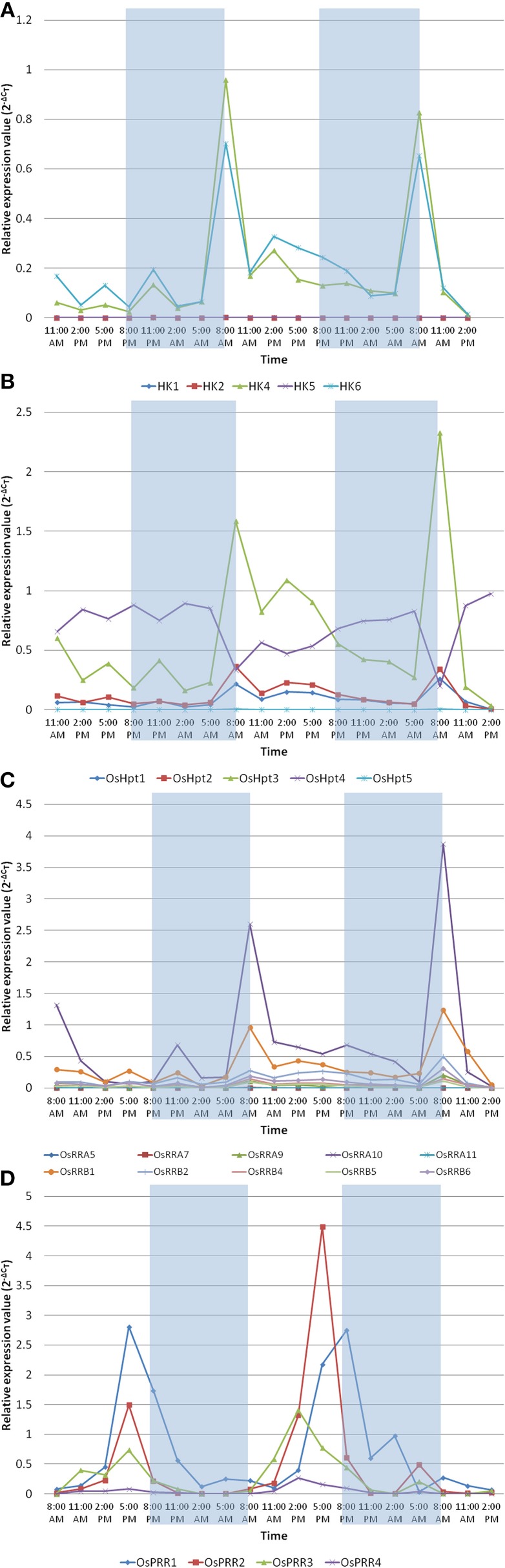
**Expression analysis of the representative family members of two-component systems, (A) OsHKs, (B) OsHpts, (C) OsRRAs and OsRRBs, (D) OsPRRs in seedlings of ***Oryza sativa*** L. (cv IR-64) during day and night cycles**. The rice seedlings were subjected to 12 h of dark followed by the 12 h of light period. The shaded area shows dark and non shaded area shows the light period.

The circadian clock controls many aspects of plant physiology such as flowering, photosynthesis and growth. The diurnal expression profiles of representative genes of TCS family were analyzed under conditions of 12 h light (L)/12 h dark (D) with a constant temperature 28 ± 2°C using IR64 genotype of rice at the seedling stage. Histidine kinases showed rhythmic expression in diurnal manner in response to light and dark cycle (Figure 6A). *OsHK1, OsHK2*, and *OsHK5* genes showed comparatively low expression while *OsHK4* and *OsHK6* showed comparatively high expression. *OsHK4* and *OsHK6* showed same pattern of expression, with a phase of 24 h and peak at transition period of night to light during morning. It was evident from the expression analysis that the HKs followed diurnal cycle. Similar to the rhythmic cycle of HKs, Hpts, and RRs also showed expression in diurnal cycle (Figures 6B,C). Among the Hpts, *OsHpt5* showed comparatively low expression than other members of the family. Changes in expression levels of *Hpts* also coincide with dark to light transition in diurnally regulated manner. Level of mRNAs of all *OsHpts* genes except *OsHpt4* oscillated during the 24 h cycle of light-dark, peaking in the morning. *OsHpt3* expression oscillated with higher amplitude in comparison to that of other *Hpts*.

Among the RRs, A type RR- *OsRRA10* and B type RR- *OsRRB1* showed comparatively high expression (Figure 6C). *OsPRR1, OsPRR2, OsPRR3* and *OsPRR4* belong to pseudo-response regulator family. Expression of each member of this family rhythmically oscillated in the given 24 h period (Figure 6D). Interestingly, the level of each mRNA reached its maximum at a distinctive time. *OsPRR1* showed evening specific peak. mRNA of members of PRR family started accumulating after dawn sequentially at 3 h intervals in the order of *OsPRR3*→*OsPRR4*→*OsPRR2*→*OsPRR1. OsPRR2*>*OsPRR1*>*OsPRR3*>*OsPRR4* is the order of their expression amplitude.

In summary, most of the considered TCS members except pseudo-response regulators, showed similar pattern of expression, with a phase of 24 h and peak at transition period of night to light during morning.

## Discussion

TCS is considered as one of the most crucial signal transduction system in plants. Evidence suggest that TCS pathways are involved in sensing the environmental stimuli, ethylene signaling, light perception, circadian rhythm and cytokinin-dependent processes which include shoot and root development, vascular differentiation and leaf senescence (Hwang et al., 2002; Kakimoto, 2003; Tran et al., 2010). Cytokinin signaling has been associated with the variety of stress response (Hare et al., 1997). Histidine kinase of the TCS is known to function as an oxidative stress sensor (Singh, 2000). *ERS1* gene provides ethylene sensitivity to the plants. Analysis has shown the accumulation of ERS1 in the leaves of *Nicotiana tabacum* L. on exogenous ethylene treatment, while transcripts were observed in root, shoot, and leaf of the plant (Terajima et al., 2001). MPSS analysis also showed the accumulation of transcripts in roots and leaves in *Arabidopsis*. Cytokinin receptor, CRE1 transcripts were observed to accumulate in root tissues. Recent analysis has shown that CRE1 cytokinin pathway is differentially recruited depending on the root environmental conditions in *Medicago truncatula* (Laffont et al., 2015). Further, the expression of *AHK2, AHK*3, and *AHK*4 was observed in several organs of the plant species (Ueguchi et al., 2001). In *Arabidopsis*, various histidine kinases namely AHK2, AHK3, and CRE1 (cytokinin response1/AHK4) are considered as principle cytokinin receptors. Mutant analysis of these cytokinin receptors confirmed their role in response toward low water potential and salt stress (Kumar and Verslues, 2015). Expression analysis showed little or no change in the expression of *AHK2* and *CRE1* genes in various abiotic stresses in both root and shoot tissues. In the present analysis, *AHK3* showed two fold expression at 12 h of salt stress. *AHK2* and *AHK3* were found to be negatively controlling osmotic stress responses in *Arabidopsis*. *CRE1* also negatively regulates osmotic stress in the presence of cytokinin (Tran et al., 2007). It was found that cold stress did not significantly induce *AHK2* and *AHK3* expression which indicates that these proteins may mediate cold temperatures for A-type RR expression (Jeon et al., 2010). Our expression analysis of the *AHK2* and *AHK3* genes in cold stress in both root and shoot tissues corroborated with the earlier result. Previously, *AHP1* was shown to be expressed in roots; *AHP2* and *AHP3* were found to express more in roots, stems, leaves, flowers, and siliques (Suzuki et al., 1998; Hradilova and Brzobohaty, 2007). Our MPSS data analysis showed the accumulation of *AHP2* and *AHP3* transcripts in the root, silique and inflorescent tissues. Analysis shows that Hpt proteins in *Arabidopsis* namely *AHP2, AHP3*, and *AHP5* control the response toward drought stress in negative and redundant manner. Also, the downregulated expression of these genes was observed under dehydrating conditions which is assumed to be due to the stress induced reduction of the endogenous cytokinin levels (Nishiyama et al., 2013). Analysis using microarray also showed downregulated expression of these genes under various abiotic stress pertaining to dehydrating conditions such as osmotic and salt stress. Recently, a knock-down analysis of two histidine phosphotransfer (*OsHpt2* and *OsHpt3*) via RNA interference (RNAi) showed that OsHpts function as positive regulators of the cytokinin signaling pathway and play different roles in salt and drought tolerance in rice (Sun et al., 2014). A 1.5-fold expression of these genes in the various rice genotypes in microarray, as reported in the present analysis, also supports the earlier results. Earlier, type-A response regulator genes in rice were shown to have an overlapping/differential expression patterns in various organs and in response to light (Jain et al., 2006). Previously, under short day conditions, B-type RR, *Ehd1* (Early heading date 1) from rice has been shown to be a floral inducer (Doi et al., 2004). In *Arabidopsis*, B-type RRs are involved in cytokinin and ethylene signaling (Hwang et al., 2002) while in rice they are involved in developmental and environmental signals mediated by light, cytokinin, and ethylene (Doi et al., 2004). Multiple A type ARRs were found to be upregulated by cold stress (Argueso et al., 2009). The upregulation of A type RR was also observed in the expression analysis in both *Arabidopsis* and rice. In *Arabidopsis*, the expression of *ARR4* and *ARR5* is found to be induced by the low temperature, dehydration and high salinity (Urao et al., 1998). Triple mutant analysis among the pseudo-RRs (APRRs) showed *APRR5, APRR7* and *APRR9* as the negative regulators in the abiotic stress conditions (Nakamichi et al., 2009).

All the organisms have a natural time keeping mechanism popularly known as circadian clocks that is used for the coordination of the physiology of organism with its surrounding environment. In plants, circadian clocks have been shown to play a major role in regulating numerous stress and growth response mechanism (Dodd et al., 2005). Regulation of signaling of phytohormones like auxin and ABA by circadian clock has been reported (Covington and Harmer, 2007; Seung et al., 2012). Earlier, in *Arabidopsis*, two Myb-related transcription factors, *circadian clock associated* (*CCA1*) and *late elongated hypocotyls* (*LHY*) have been shown to induce the expression of *PRR7* and *PPR9* in circadian rhythm (in morning cycles) and *PRR1* (in evening cycles) which also, in turn bind and repress the expression of the formers (Alabadí et al., 2001; Nakamichi et al., 2010). Earlier, *prr9/prr7/prr5* triple mutant analysis revealed the molecular link between metabolism and the circadian clock (Fukushima et al., 2009). In *Arabidopsis*, pseudoresponse regulators have been shown to be involved in circadian rhythms, control of flowering time and also photo-sensory signal transduction (Devlin and Kay, 2001; Mouradov et al., 2002). Our data show that the expression of *OsPrr* genes is under diurnal control in indica rice IR64. Murakami et al. (2003) did similar analysis in japonica *O. sativa* (var Nipponbare) and observed similar results. It again indicates both the dicotyledonous (e.g., *Arabidopsis thaliana*) and monocotyledonous (e.g., *Oryza sativa*) plants might share the evolutionarily conserved molecular mechanism underlying the circadian rhythm. Our expression analysis of representative members of the TCS family showed that not only PRRs, but also other members like HKs, Hpts and RRs are also regulated by the diurnal clock. The TCS members have been shown to play a major role in the abiotic stress response mechanism. The result provides a crucial input related to molecular link between abiotic stress response and diurnal clock.

## Conclusions

The progress made over a decade has enhanced our understanding about the two-component signaling system and the crucial role played by its members in perceiving environmental stimuli. Even though the members of the TCS system have been characterized in many plant species but their functional involvement in various environmental stress conditions is still a conundrum. The current analysis has assembled all the expression data for all the TCS members, in order to understand their functional complexity. Further, MPSS data analysis presented an overview of the transcript abundance of the TCS members in various plant tissues under various stress conditions. Expression analysis suggest that rice involves more number of TCS members (HKs, Hpts, and RRs) in these responses, despite having comparable number of genes with respect to *Arabidopsis* (Table 2). Also, the diurnal and rhythmic expression of the TCS gene family members in response to the day and night cycle provides a crucial information about the complexities of the process that are regulated by various TCS members in response to various abiotic stress conditions. The analysis presented in this study provides interesting insights about the functional involvement of the TCS members in growth and stress response in plants.

**Table 2:** **Table showing genes of the TCS family which were found to be altered significantly (≥1.5 fold; upregulation/downregulation) under various abiotic stress conditions**.

	***Oryza sativa***		***Arabidopsis thaliana***	
	**Up-regulated**	**Down-regulated**	**Up-regulated**	**Down-regulated**
HK	OsHK5 (1), OsHK6 (1), OsERS2 (1), OsPHYA (1), OsHK2 (4), OsETR3 (3)	OsHK4 (4), OsPHYB (2), OsPHYC (3), OsERS1 (1), OsHK5 (2), OsHK6 (3), OsERS2 (2), OsPHYA (2), OsHK2 (4), OsETR3 (2)	CK12 (6), CK11 (12), ETR2(1)	AHK1 (4), CRE1 (1), AHK2 (1), CK12 (2), CK11 (13), ETR2 (1)
Hpt	OsHpt1 (3), OsHpt2 (1), OsHpt4 (3), OsHpt5 (1)	OsHpt1 (2), OsHpt3 (1), OsHpt2 (2), OsHpt4 (5), OsHpt5 (4)	AHP6 (1), AHP4 (23)	AHP5 (1), AHP1 (1), AHP3 (1), AHP6 (8), AHP4 (8)
RR	OsRRA1 (5), OsRRA3 (3), OsRRA9 (1), OsRRA10 (2), OsPRR3 (5), OsRRB4 (1), OsRRA4 (1), OsRRA14 (1), OsRRA16 (1), OsRRB2 (1), OsRRA19 (2), OsRRA5 (2), OsRRA15 (5), OsRRB5 (2), OsRRA18 (3), OsPRR4 (5), OsRRB6 (1), OsETR2 (1), OsRRB3 (5), OsRRA22 (3), OsRRA13 (3), OsRRA6 (4), OsRRA12 (5), OsETR4 (2), OsRRA7 (2), OsRRA17 (5), OsRRB1 (7), OsPRR5 (11), OsPRR1 (12), OsPRR2 (11), OsRRB7 (3)	OsRRA1(14), OsRRA3(12), OsRRA9(10), OsRRA10(11), OsPRR3(12), OsRRB4(6), OsRRA4(6), OsRRA14(5), OsRRA16(3), OsRRB2(3), OsRRA19(1), OsRRA5(1), OsRRA15(1), OsRRB5(1), OsRRA18(1), OsPRR4(1), OsRRB6(1), OsETR2(1), OsRRB3(1), OsRRA22, OsRRA13(1), OsRRA6(1), OsRRA12(1), OsETR4(1), OsRRA7(1), OsRRA17(1), OsRRB1(1), OsPRR5(1), OsPRR1(1), OsPRR2(1)	APRR9 (22), APRR4 (12), ARR23 (17), ARR7 (5), ARR17 (9), ARR6 (6), ARR18 (3), ARR19 (6), ARR11 (1), ARR3 (2), ARR22 (1), APRR3 (1), ARR16 (3), ARR5 (7), APRR5 (7), ARR15 (4), APRR8 (2), ARR2 (1), APRR1 (3), APRR7 (5), ARR21 (10)	APRR9 (2), APRR4 (4), ARR23 (9), ARR7(11), ARR17(17), ARR6 (29), ARR18 (15), ARR19 (20), ARR11 (9), ARR3 (6), ARR22 (5), APRR3 (3), ARR16 (4), ARR5 (9), APRR5 (2), ARR15 (2), ARR9 (4), ARR1 (1), APRR2 (2), ARR10 (2), ARR8 (5), ARR4 (8), ARR14 (2), ARR21 (11)

*The numbers in parenthesis with the gene name shows the number of conditions in which their alteration was observed*.

## Author contributions

AP and SLS-P conceived the idea and designed the experiments. PS and HG did the real time PCR work and its analysis. AS and HK performed the MPSS and microarray database analysis and wrote the manuscript. AP and SLS-P edited the manuscript. All the authors approved the final manuscript.

### Conflict of interest statement

The authors declare that the research was conducted in the absence of any commercial or financial relationships that could be construed as a potential conflict of interest.
