# Supplementary material for: Tissue specific and abiotic stress regulated transcription of histidine kinases in plants is also influenced by diurnal rhythm
Source: Front Plant Sci. 2015 Sep 11;6:711. doi: 10.3389/fpls.2015.00711 (PMC4566072; doi:10.3389/fpls.2015.00711)
Supplement: Supplementary file 1 [file Table1.DOCX]

***Supplementary Material***

**Tissue specific and abiotic stress regulated transcription of histidine kinases in plants is also influenced by diurnal rhythm**

**Anupama Singh^1^, Hemant Ritturaj Kushwaha^2^, Praveen Soni^3^, Himanshu Gupta^3^, Sneh Lata Singla-Pareek^4^, Ashwani Pareek^3*^**

^1^School of Computational and Integrative Sciences, Jawaharlal Nehru University, New Delhi, India

^2^Synthetic Biology and Biofuels Group, International Centre for Genetic Engineering and Biotechnology, New Delhi, India

^3^Stress Physiology and Molecular Biology Laboratory, School of Life Sciences, Jawaharlal Nehru University, New Delhi, India

^4^Plant Molecular Biology Group, International Centre for Genetic Engineering and Biotechnology, New Delhi, India

*** Correspondence:** Professor Ashwani Pareek, Stress Physiology and Molecular Biology Laboratory, School of Life Sciences, Jawaharlal Nehru University, New Delhi, 110067, India

[ashwanip@mail.jnu.ac.in](mailto:ashwanip@mail.jnu.ac.in)

**Supplementary Table 1:** Microarray data used in the analysis in GEO database for *O. sativa*

| **GEO ID** | **Pubmed ID** | **Genotype** | **sub genotype** | **Growth stage/tissue** | **Stress type** |
| --- | --- | --- | --- | --- | --- |
| GSE3053 | 16183841 | indica | FL478 | vegetative stage | Salinity |
| GSE4438 | 17160619 | indica | IR63731 | Panicle initiation Stage | Salinity |
| GSE4438 | 17160619 | japonica | Agami | Panicle Initiation Stage | Salinity |
| GSE6901 | 19788421 | indica | IR64 | 7 days old rice seedling | Salinity |
| GSE6901 | 19788421 | indica | IR64 | 7 days old rice seedling | Cold |
| GSE6901 | 19788421 | indica | IR64 | 7 days old rice seedling | Drought |
| GSE14275 |  | Japonica | ZhongHua 11 | 14-day-old seedling | heat shock in rice seedlings |
| GSE14403 | 20924028 | indica | FL478 | 22 day germination Root | Salinity |
| GSE16108 | 20602115 | indica | MI48 | Seedling 10 days | Salinity |
| GSE13735 | 19706179 | indica | FL478 | root tip | Salinity |
| GSE21651 | Unpublished, 2010 | indica | IR64 | Two weeks old seedlings, leaf | Drought |
| GSE21651 | Unpublished, 2010 | indica | VSR | two weeks old seedlings, leaf | Salinity |
| GSE24048 | Unpublished, 2010 | Japonica | Azucena | Rice leaf from plant  drought for 24 days  grown in a field | Drought |
| GSE24048 | Unpublished, 2010 | indica | Bala | Rice leaf from plant  drought for 24 days  grown in a field | Drought |
| GSE26280 | 21406116 | indica | DK151 | Tillering stage leaf | Drought |
| GSE26280 | 21406116 | indica | DK151 | Tillering stage Root | Drought |
| GSE26280 | 21406116 | indica | DK151 | Panicle Elongation stage leaf | Drought |
| GSE26280 | 21406116 | indica | DK151 | Panicle Elongation stage Root | Drought |
| GSE26280 | 21406116 | indica | DK151 | Booting Stage leaf | Drought |
| GSE26280 | 21406116 | indica | DK151 | Booting stage young panicle | Drought |
| GSE37161 | 22963580 | Japonica | japonica | Root tip | Nutrition Deficiency 6hrs |
| GSE37161 | 22963580 | Japonica | japonica | Root tip | Nutrition Deficiency 3hrs |
| GSE37161 | 22963580 | Japonica | japonica | Root tip | Nutrition Deficiency 5 days |
| GSE37940 | 22953761 | Japonica | K354 | 3leaf seeding stage | cold stress 2hr |
| GSE37940 | 22953761 | Japonica | K354 | 3leaf seeding stage | cold stress 6hr |
| GSE37940 | 22953761 | Japonica | K354 | 3leaf seeding stage | cold stress 12hr |
| GSE37940 | 22953761 | Japonica | K354 | 3leaf seeding stage | cold stress 24hr |
| GSE37940 | 22953761 | Japonica | K354 | 3leaf seeding stage | cold stress 48hr |
| GSE38023 | 22912843 | Japonica | Li-Jiang-Xin-Tuan-Hei-Gu  (LJX-  THG) | Leaf S3 stage | cold stress 2hr |
| GSE38023 | 22912843 | Japonica | Li-Jiang-Xin-Tuan-Hei-Gu  (LJX-  THG) | Leaf S3 stage | cold stress 8hr |
| GSE38023 | 22912843 | Japonica | Li-Jiang-Xin-Tuan-Hei-Gu  (LJX-  THG) | Leaf S3 stage | cold stress 24hr |
| GSE38023 | 22912843 | Japonica | Li-Jiang-Xin-Tuan-Hei-Gu  (LJX-  THG) | Leaf S3 stage | cold stress 48hr |
| GSE38023 | 22912843 | indica | IR29 | Leaf S3 stage | cold stress 2hr |
| GSE38023 | 22912843 | indica | IR29 | Leaf S3 stage | cold stress 8hr |
| GSE38023 | 22912843 | indica | IR29 | Leaf S3 stage | cold stress 24hr |
| GSE38023 | 22912843 | indica | IR29 | Leaf S3 stage | cold stress 48hr |
| GSE38023 | 22912843 | indica | IR29 | Leaf S3 stage | cold stress 24hr |
| GSE41103 | Unpublished, 2012 | Japonica | M202 | 18 day old seedlings  shoot | submergence  stress for 1 day |
| GSE41103 | Unpublished, 2012 | Japonica | M202 | 18 day old seedlings  shoot | submergence  stress for 6 day |
| GSE44250 | 23922980 | indica | IR64 | Seedling | Potassium minus  KM medium  stress 5 days |
| GSE44250 | 23922980 | indica | IR64 | Seedling | Resumed K in  KM samples for 6hr |
| GSE45724 | 23685753 | Japonica | Jijing88 | 7 day old seedling  shoot | Alkali salt for 24  hr |
