# Supplementary material for: Tissue specific and abiotic stress regulated transcription of histidine kinases in plants is also influenced by diurnal rhythm
Source: Front Plant Sci. 2015 Sep 11;6:711. doi: 10.3389/fpls.2015.00711 (PMC4566072; doi:10.3389/fpls.2015.00711)
Supplement: Supplementary file 2 [file Table2.DOCX]

***Supplementary Material***

**Tissue specific and abiotic stress regulated transcription of histidine kinases in plants is also influenced by diurnal rhythm**

**Anupama Singh^1^, Hemant Ritturaj Kushwaha^2^, Praveen Soni^3^, Himanshu Gupta^3^, Sneh Lata Singla-Pareek^4^, Ashwani Pareek^3*^**

**^1^School of Computational and Integrative Sciences, Jawaharlal Nehru University, New Delhi, India**

**^2^Synthetic Biology and Biofuels Group, International Centre for Genetic Engineering and Biotechnology, New Delhi, India**

**^3^Stress Physiology and Molecular Biology Laboratory, School of Life Sciences, Jawaharlal Nehru University, New Delhi, India**

**^4^Plant Molecular Biology Group, International Centre for Genetic Engineering and Biotechnology, New Delhi, India**

*** Correspondence:** Professor Ashwani Pareek, Stress Physiology and Molecular Biology Laboratory, School of Life Sciences, Jawaharlal Nehru University, New Delhi, 110067, India

[ashwanip@mail.jnu.ac.in](mailto:ashwanip@mail.jnu.ac.in)

**Supplementary Table 2:** Lists of primers used in this study.

| Gene | Primer | Nucleotide sequence |
| --- | --- | --- |
| *OsHK1* | LOC_Os06g44410FP | GCAGGCATGGACTCCTACATATC |
|  | LOC_Os06g44410RP | TGCTGCAGGCATTCTTTAATATTT |
| *OsHK2* | LOC_Os06g08450FP | CGCGAATGGATGGCTATGA |
|  | LOC_Os06g08450RP | CGAATTCCGTAGCGGCTTT |
| *OsHK4* | LOC_Os03g50860FP | ACAGCTCCCACGATTTTTCG |
|  | LOC_Os03g50860RP | CACCCTGCAAACATGTCTTCTG |
| *OsHK5* | LOC_Os10g21810FP | CGTTTCCAACAGCAAAACAGAA |
|  | LOC_Os10g21810RP | GGAAATACTCGTCGTTTCAGACAG |
| *OsHK6* | LOC_Os02g50480FP | TTGTGCCATACTGTCAAGTTCCA |
|  | LOC_Os02g50480RP | TGGTGGTCGAGCACTTTTTTT |
| *OsHpt1* | LOC_ Os01g54050FP | GAGTGCATGTCGTTCAGGGATA |
|  | LOC_ Os01g54050RP | AGACCTCATGCAACCTTCAACA |
| *OsHpt2* | LOC_ Os08g44350FP | AGCTTTGGCACCTTCATTACTGA |
|  | LOC_ Os08g44350RP | GAAACGGTTGTCCCCATCAC |
| *OsHpt3* | LOC_ Os09g39400FP | GCGCGTGTGCAGAATCAA |
|  | LOC_ Os09g39400RP | GTTATGAGCTCAAAGTTGGAAGC |
| *OsHpt4* | LOC_ Os05g09410FP | TGAAAAGTTTTTGTGCCAATCAGA |
|  | LOC_ Os05g09410RP | TGCAATCCAAACACAACAAAAAG |
| *OsHpt5* | LOC_ Os05g44570FP | GCGACTAGGCTGGTACACAAGA |
|  | LOC_ Os05g44570RP | GGGTTTTCACCGTAGTGGCATA |
| *OsRRA5* | LOC_Os07g26720FP | GAGAACGTGCCCACAAGGAT |
|  | LOC_Os07g26720RP | CACGCTTGATCGGTGAAGAG |
| *OsRRA7* | LOC_Os02g58350FP | AAGAGGAGTAGCAATCGCACAAT |
|  | LOC_Os02g58350RP | TCTGATCTTCGTCTTGTTGTCGTT |
| *OsRRA9* | LOC_Os04g36070FP | GGCTGCAACTGGCAAAGC |
|  | LOC_Os04g36070RP | TGCTGCTCTTTCAGTGCAGAA |
| *OsRRA10* | LOC_Os02g35180FP | CTCAGTGGCATAGCGTGGATT |
|  | LOC_Os02g35180RP | AGCTGCACTCTTGCTTGATGAA |
| *OsRRA13* | LOC_Os08g28900FP | TCAAATTGGGCTTGTTTTTACTCA |
|  | LOC_Os08g28900RP | AAGCAATTACAACCGGGAGATG |
| *OsRRA15* | LOC_Os04g13480FP | CCAACAGCATGGAAGAGTTCAA |
|  | LOC_Os04g13480RP | GCTTCAGCTTGGGCACACA |
| *OsRRA18* | LOC_Os05g32890FP | TCCTCGGTTCACTTCTACAGAATTT |
|  | LOC_Os05g32890RP | ATGAGAGAATGCAGTAAGTGAAAAG |
| *OsRRB1* | LOC_Os03g12350FP | TGACAGCATGTCGAATCTTGGT |
|  | LOC_Os03g12350RP | TCACATGAGGCTAAATAATCCAAAGT |
| *OsRRB2* | LOC_Os02g08500FP | CCAGCCTCCATTTCAATCATTAG |
|  | LOC_Os02g08500RP | CGACACCGCGAATGCA |
| *OsRRB3* | LOC_Os06g43910FP | GACACAAGCCCAGCAGAGATG |
|  | LOC_Os06g43910RP | AGAACTGATGTTCGAGTCACTACCA |
| *OsRRB4* | LOC_Os06g08440FP | TGCCTGATCTGTGCTACTTCATC |
|  | LOC_Os06g08440RP | GATTACAAGCCACTACTGTCCCTAGA |
| *OsRRB5* | LOC_Os02g55320FP | CCAACTCGACCTAGAAAGAGAGACA |
|  | LOC_Os02g55320RP | CGAGGAAGAAGAAACTGACAGTAAG |
| *OsRRB6* | LOC_Os01g67770FP | CTGCCGCCCTTCTTTCG |
|  | LOC_Os01g67770RP | TGGGAACATACCAAAGAACATCAT |
| *OsPRR1* | LOC_Os02g40510FP | CATCCACTCGCTCACACACAA |
|  | LOC_Os02g40510RP | CAGTGATGGATATGCCACAAGTTC |
| *OsPRR2* | LOC_Os09g36220FP | TGCTGTGCCACGGTTATCC |
|  | LOC_Os09g36220RP | GGCAATCATACTTTCACTCTTCAAAC |
| *OsPRR3* | LOC_Os03g17570FP | CCAACATGGCATCTGCTTTG |
|  | LOC_Os03g17570RP | TGCTTTCAGCTATGGAATGAATTG |
| *OsPRR4* | LOC_Os07g49460FP | CGAGAGTGCTCTGCCCTGTAG |
|  | LOC_Os07g49460RP | GACCAGTGACCCAACAAACACA |
