# Supplementary material for: Tissue specific and abiotic stress regulated transcription of histidine kinases in plants is also influenced by diurnal rhythm
Source: Front Plant Sci. 2015 Sep 11;6:711. doi: 10.3389/fpls.2015.00711 (PMC4566072; doi:10.3389/fpls.2015.00711)
Supplement: Supplementary file 3 [file Table3.DOCX]

***Supplementary Material***

**Tissue specific and abiotic stress regulated transcription of histidine kinases in plants is also influenced by diurnal rhythm**

**Anupama Singh^1^, Hemant Ritturaj Kushwaha^2^, Praveen Soni^3^, Himanshu Gupta^3^, Sneh Lata Singla-Pareek^4^, Ashwani Pareek^3*^**

^1^School of Computational and Integrative Sciences, Jawaharlal Nehru University, New Delhi, India

^2^Synthetic Biology and Biofuels Group, International Centre for Genetic Engineering and Biotechnology, New Delhi, India

^3^Stress Physiology and Molecular Biology Laboratory, School of Life Sciences, Jawaharlal Nehru University, New Delhi, India

^4^Plant Molecular Biology Group, International Centre for Genetic Engineering and Biotechnology, New Delhi, India

*** Correspondence:** Professor Ashwani Pareek, Stress Physiology and Molecular Biology Laboratory, School of Life Sciences, Jawaharlal Nehru University, New Delhi, 110067, India

[ashwanip@mail.jnu.ac.in](mailto:ashwanip@mail.jnu.ac.in)

**Supplementary Table 3a:** Putative HK and related proteins in *Arabidopsis thaliana*

| **Gene** | **Proteins** | **Locus** | **Coordinates** | **AA** |
| --- | --- | --- | --- | --- |
| *AHK1* | AHK1 | AT2G17820.1 | 7743133-7748013 | 1027 |
| *AHK2* | AHK2 | AT5G35750.1 | 13911743-13916337 | 1176 |
| *AHK3* | AHK3 | AT1G27320.1 | 9487780-9492027 | 1207 |
| *CK11* | CK11 | AT2G47430.1 | 19459167-19463122 | 1122 |
| *CK12* | CK12 | AT5G10720.1 | 3386835-3390541 | 922 |
| *CRE1* | CRE1a | AT2G01830.2 | 363332-368016 | 1080 |
|  | CRE1b | AT2G01830.3 | 363332-367429 | 1057 |
|  | CRE1c | AT2G01830.1 | 363332-367429 | 1057 |
| *ETR1* | ETR1 | AT1G66340.1 | 24734698-24737366 | 738 |
| *ETR2* | ETR2 | AT3G23150.1 | 8255156-8257560 | 773 |
| *ERS1* | ERS1 | AT2G40940.1 | 17084635-17086819 | 613 |
| *EIN4* | EIN4a | AT3G04580.1 | 1235576-1237965 | 766 |
|  | EIN4b | AT3G04580.2 | 1235576-1237965 | 766 |
| *PHYA* | PHYAa | AT1G09570.1 | 3095498-3099216 | 1122 |
|  | PHYAb | AT1G09570.2 | 3095498-309889 | 1014 |
| *PHYB* | PHYB | AT2G18790.1 | 8140079-8144151 | 1172 |
| *PHYC* | PHYC | AT5G35840.1 | 14008049-14011619 | 1111 |
| *PHYD* | PHYD | AT4G16250.1 | 9195602-9199486 | 1164 |
| *PHYE* | PHYE | AT4G18130.1 | 10042312-10045948 | 1112 |

**Table 3b:** Putative HK and related proteins in *Oryza sativa*

| **Gene** | **Proteins** | **TIGR id** | **Locus** | **Coordinates** | **AA** |
| --- | --- | --- | --- | --- | --- |
| *OsHK1* | OsHK1 | 13106.m04668 | LOC_Os06g44410.1 | 26807861 - 26813600 | 969 |
| *OsHK2* | OsHK2 | 13106.m00882 | LOC_Os06g08450.1 | 4150976 - 4142851 | 1049 |
| *OsHK3* | OsHK3a | 13101.m07585 | LOC_Os01g69920.1 | 40409615 - 40403715 | 1024 |
|  | OsHK3b | 13101.m07586 | LOC_Os01g69920.2 | 40409615 - 40403715 | 867 |
|  | OsHK3c | 13101.m14973 | LOC_Os01g69920.3 | 40409615 - 40403715 | 939 |
| *OsHK4* | OsHK4 | 13103.m05531 | LOC_Os03g50860.1 | 29052318 - 29058566 | 1014 |
| *OsHK5* | OsHK5a | 13110.m01810 | LOC_Os10g21810.1 | 11203063 - 11192316 | 1187 |
|  | OsHK5b | 13110.m01811 | LOC_Os10g21810.2 | 11203063 - 11192316 | 609 |
| *OsHK6* | OsHK6 | 13102.m05729 | LOC_Os02g50480.1 | 30823545 - 30829508 | 998 |
| *OsHKL1* | OsHKL1 | 13106.m03947 | LOC_Os06g38130.1 | 22576952 - 22578191 | 378 |
| *OsETR3* | OsETR3a | 13102.m06645 | LOC_Os02g57530.1 | 35251393 - 35256043 | 837 |
|  | OsETR3b | 13102.m06644 | LOC_Os02g57530.2 | 35251393 - 35256043 | 771 |
|  | OsETR3c | 13102.m06643 | LOC_Os02g57530.3 | 35251393 - 35256043 | 771 |
|  | OsETR3d | 13102.m06646 | LOC_Os02g57530.4 | 35251393 - 35256043 | 607 |
| *OsERS1* | OsERS1a | 13103.m05366 | LOC_Os03g49500.1 | 28174250 - 28169807 | 637 |
|  | OsERS1b | 13103.m05367 | LOC_Os03g49500.2 | 28173984 - 28169807 | 637 |
| *OsERS2* | OsERS2a | 13105.m00676 | LOC_Os05g06320.2 | 3237383 - 3233010 | 519 |
|  | OsERS2b | 13105.m00675 | LOC_Os05g06320.3 | 3235670 - 3233010 | 360 |
| *OsPHYA* | OsPHYAa | 13103.m05548 | LOC_Os03g51030.1 | 29176112 - 29168142 | 1129 |
|  | OsPHYAb | 13103.m05549 | LOC_Os03g51030.2 | 29176095 - 29168142 | 1129 |
|  | OsPHYAc | 13103.m05550 | LOC_Os03g51030.3 | 29176095 - 29168142 | 1129 |
| *OsPHYB* | OsPHYB | 13103.m02346 | LOC_Os03g19590.1 | 11020091 - 11028228 | 1172 |
| *OsPHYC* | OsPHYCa | 13103.m05925 | LOC_Os03g54084.1 | 31004724 - 31009782 | 1138 |

**Table 3c:** Putative Hpt proteins in *Arabidopsis thaliana*

| **Gene** | **Proteins** | **Locus** | **Coordinates** | **AA** |
| --- | --- | --- | --- | --- |
| *AHP1* | AHP1 | AT3G21510.1 | 7578432-7579537 | 154 |
| *AHP2* | AHP2a | AT3G29350.1 | 11264379-11265408 | 156 |
|  | AHP2b | AT3G29350.2 | 11264682-11265408 | 114 |
| *AHP3* | AHP3 | AT5G39340.1 | 15748941-15750248 | 155 |
| *AHP4* | AHP4a | AT3G16360.1 | 5554484-5555367 | 127 |
|  | AHP4b | AT3G16360.2 | 5554351-5555518 | 145 |
| *AHP5* | AHP5 | AT1G03430.1 | 848159-849235 | 157 |
| *AHP6* | AHP6a | AT1G80100.1 | 30133818-30134652 | 154 |
|  | AHP6b | AT1G80100.2 | 30133818-30134652 | 132 |

**Table 3d:** Putative Hpt proteins in *Oryza sativa*

| **Gene** | **Proteins** | **TIGR id** | **Locus** | **Coordinates** | **AA** |
| --- | --- | --- | --- | --- | --- |
| *OsHpt1* | OsHpt1 | 13101.m05676 | LOC_Os01g54050.1 | 31092032 - 31094953 | 152 |
| *OsHpt2* | OsHpt2 | 13108.m04810 | LOC_Os08g44350.1 | 27904573 - 27909555 | 148 |
| *OsHpt3* | OsHpt3a | 13109.m03948 | LOC_Os09g39400.1 | 22653849 - 22657046 | 150 |
|  | OsHpt3b | 13109.m03947 | LOC_Os09g39400.2 | 22653849 - 22657046 | 119 |
| *OsHpt4* | OsHpt4 | 13105.m01049 | LOC_Os05g09410.1 | 5280638 - 5278198 | 152 |
| *OsHpt5* | OsHpt5a | 13105.m04714 | LOC_Os05g44570.1 | 25931525 - 25927984 | 153 |
|  | OsHpt5b | 13105.m04715 | LOC_Os05g44570.3 | 25935287 - 25927984 | 97 |

**Table 3e:** Putative RR proteins in *Arabidopsis thaliana*

| **Gene** | **Proteins** | **Locus** | **Coordinates** | **AA** |
| --- | --- | --- | --- | --- |
| **Response regulator like proteins** | | | | |
| *APRR1* | APRR1 | AT5G61380.1 | 24675540-24678176 | 618 |
| *APRR2* | APRR2a | AT4G18020.1 | 10003738-10006682 | 535 |
|  | APRR2b | AT4G18020.2 | 10003738-10006682 | 535 |
|  | APRR2c | AT4G18020.3 | 10003738-10006682 | 535 |
|  | APRR2d | AT4G18020.4 | 10003991-10006682 | 487 |
|  | APRR2e | AT4G18020.5 | 10003991-10006682 | 487 |
|  | APRR2f | AT4G18020.6 | 10003738-10006682 | 535 |
| *APRR3* | APRR3a | AT5G60100.1 | 24198215-24200502 | 495 |
|  | APRR3b | AT5G60100.3 | 24198215-24200502 | 495 |
|  | APRR3c | AT5G60100.2 | 24198215-24200502 | 522 |
| *APRR4* | APRR4 | AT5G49240.1 | 19962934-19964351 | 292 |
| *APRR5* | APRR5 | AT5G24470.1 | 8356204-8358873 | 667 |
| *APRR6* | APRR6 | AT1G68210.1 | 25565983-25569302 | 755 |
| *APRR7* | APRR7 | AT5G02810.1 | 638283-641461 | 727 |
| *APRR8* | APRR8 | AT4G00760.1 | 327236-328955 | 336 |
| *APRR9* | APRR9 | AT2G46790.1 | 19232874-19234901 | 468 |
| **B type** | | | | |
| *ARR1* | ARR1a | AT3G16857.1 | 5756113-5758853 | 669 |
|  | ARR1b | AT3G16857.2 | 5756113-5759139 | 690 |
| *ARR2* | ARR2 | AT4G16110.1 | 9112979-9115785 | 664 |
| *ARR10* | ARR10 | AT4G31920.1 | 15444290-15446766 | 552 |
| *ARR11* | ARR11 | AT1G67710.1 | 25376994-25378905 | 521 |
| *ARR12* | ARR12 | AT2G25180.1 | 10724490-10726961 | 596 |
| *ARR13* | ARR13 | AT2G27070.1 | 11555781-11560215 | 575 |
| *ARR14* | ARR14 | AT2G01760.1 | 333041-334514 | 382 |
| *ARR18* | ARR18 | AT5G58080.1 | 23501785-23504099 | 618 |
| *ARR19* | ARR19a | AT1G49190.1 | 18191342-18193598 | 608 |
|  | ARR19b | AT1G49190.2 | 18191342-18193598 | 622 |
| *ARR20* | ARR20 | AT3G62670.1 | 23176556-23177922 | 352 |
| *ARR21* | ARR21 | AT5G07210.1 | 2252237-2256018 | 621 |
| *ARR23* | ARR23 | AT5G62120.1 | 24946955-24948225 | 145 |
| **A-type** | | | | |
| *ARR3* | ARR3 | AT1G59940.1 | 22065894-22066895 | 231 |
| *ARR4* | ARR4 | AT1G10470.1 | 3442624-3443759 | 259 |
| *ARR5* | ARR5 | AT3G48100.1 | 17759112-17760740 | 184 |
| *ARR6* | ARR6 | AT5G62920.1 | 25252745-25254158 | 186 |
| *ARR7* | ARR7 | AT1G19050.1 | 6577919-6579078 | 206 |
| *ARR8* | ARR8 | AT2G41310.1 | 17222280-17223536 | 225 |
| *ARR9* | ARR9 | AT3G57040.1 | 21110059-21111228 | 234 |
| *ARR15* | ARR15 | AT1G74890.1 | 28131590-28132710 | 206 |
| *ARR16* | ARR16a | AT2G40670.1 | 16970258-16971120 | 164 |
|  | ARR16b | AT2G40670.2 | 16970258-16971120 | 165 |
| *ARR17* | ARR17 | AT3G56380.1 | 20905480-20906368 | 153 |
| *ARR22* | ARR22a | AT3G04280.1 | 1130138-1130689 | 142 |
|  | ARR22b | AT3G04280.2 | 1130138-1130689 | 142 |
|  | ARR22c | AT3G04280.3 | 1130138-1130689 | 142 |
| *ARR24* | ARR24 | AT5G26594.1 | 9269282-9270254 | 139 |

**Table 3f:** Putative RR proteins in *Oryza sativa*

| **Gene** | **Proteins** | **TIGR id** | **Locus** | **Coordinates** | **AA** |
| --- | --- | --- | --- | --- | --- |
| *OsRRA1* | OsRRA1a | 13111.m00433 | LOC_Os11g04720.1 | 2013442 - 2015269 | 209 |
|  | OsRRA1b | 13111.m00432 | LOC_Os11g04720.2 | 2013371 - 2015405 | 202 |
| *OsRRA2* | OsRRA2 | 13112.m00431 | LOC_Os12g04500.1 | 1915856 - 1917598 | 202 |
| *OsRRA3* | OsRRA3 | 13101.m07879 | LOC_Os01g72330.1 | 41948952 - 41952388 | 233 |
| *OsRRA4* | OsRRA4 | 13104.m04411 | LOC_Os04g44280.1 | 26227217 - 26228435 | 135 |
| *OsRRA5* | OsRRA5a | 13107.m02650 | LOC_Os07g26720.1 | 15440889 - 15442180 | 207 |
|  | OsRRA5b | 13107.m09955 | LOC_Os07g26720.2 | 15440889 - 15442180 | 211 |
| *OsRRA6* | OsRRA6 | 13104.m06053 | LOC_Os04g57720.1 | 34375978 - 34377357 | 131 |
| *OsRRA7* | OsRRA7 | 13102.m06758 | LOC_Os02g58350.1 | 35690390 - 35689247 | 132 |
| *OsRRA8* | OsRRA8 | 13102.m04649 | LOC_Os02g42060.1 | 25287918 - 25286937 | 148 |
| *OsRRA9* | OsRRA9 | 13104.m03532 | LOC_Os04g36070.1 | 22027680 - 22023832 | 232 |
| *OsRRA10* | OsRRA10 | 13102.m03819 | LOC_Os02g35180.1 | 21131370 - 21128620 | 253 |
| *OsRRA11* | OsRRA11 | 13108.m02989 | LOC_Os08g28950.1 | 17714143 - 17714938 | 122 |
| *OsRRA12* | OsRRA12 | 13108.m02782 | LOC_Os08g26990.1 | 16489493 - 16490328 | 122 |
| OsRRA13 | OsRRA13 | 13108.m02985 | LOC_Os08g28900.1 | 17677910 - 17678691 | 122 |
| *OsRRA14* | OsRRA14 | 13103.m05794 | LOC_Os03g53100.1 | 30451518 - 30452854 | 128 |
| *OsRRA15* | OsRRA15 | 13104.m01238 | LOC_Os04g13480.1 | 7521476 - 7522295 | 133 |
| *OsRRA16* | OsRRA16 | 13105.m03390 | LOC_Os05g32880.1 | 19260019 - 19255491 | 514 |
| *OsRRA17* | OsRRA17 | 13104.m02660 | LOC_Os04g28120.1 | 16602471 - 16605138 | 253 |
| *OsRRA18* | OsRRA18 | 13105.m03391 | LOC_Os05g32890.1 | 19265596 - 19261580 | 369 |
| *OsRRA19* | OsRRA19 | 13108.m03741 | LOC_Os08g35670.1 | 22497789 - 22492748 | 615 |
| *OsPRR3* | OsRRA20a | 13103.m02097 | LOC_Os03g17570.4 | 9768690 - 9759479 | 746 |
|  | OsRRA20b | 13103.m02098 | LOC_Os03g17570.5 | 9768690 - 9759479 | 534 |
|  | OsRRA20c | 13103.m02099 | LOC_Os03g17570.6 | 9768690 - 9759479 | 474 |
| *OsRRA22* | OsRRA22 | 13104.m02664 | LOC_Os04g28160.1 | 16629917 - 16633083 | 381 |
| **B-type** | | | | | |
| *OsRRB1* | OsRRB1a | 13103.m01410 | LOC_Os03g12350.1 | 6512744 - 6518574 | 692 |
|  | OsRRB1b | 13103.m01412 | LOC_Os03g12350.2 | 6512744 - 6518513 | 692 |
|  | OsRRB1c | 13103.m01411 | LOC_Os03g12350.4 | 6512744 - 6518792 | 692 |
| *OsRRB2* | OsRRB2 | 13102.m00983 | LOC_Os02g08500.1 | 4578363 - 4573806 | 627 |
| *OsRRB3* | OsRRB3 | 13106.m04606 | LOC_Os06g43910.1 | 26450761 - 26454696 | 695 |
| *OsRRB4* | OsRRB4 | 13106.m00881 | LOC_Os06g08440.1 | 4137248 - 4142663 | 697 |
| *OsRRB5* | OsRRB5 | 13102.m06341 | LOC_Os02g55320.1 | 33864842 - 33859825 | 689 |
| *OsRRB6* | OsRRB6 | 13101.m07371 | LOC_Os01g67770.1 | 39391101 - 39387230 | 583 |
| *OsRRB7* | OsRRB7 | 13104.m02661 | LOC_Os04g28130.1 | 16614563 - 16618026 | 391 |
| **PRR** | | | | | |
| *OsPRR1* | OsPRR1 | 13102.m04515 | LOC_Os02g40510.1 | 24569294 - 24572560 | 519 |
| *OsPRR2* | OsPRR2a | 13109.m03560 | LOC_Os09g36220.1 | 20889843 - 20885172 | 624 |
|  | OsPRR2b | 13109.m07593 | LOC_Os09g36220.2 | 20889843 - 20885172 | 624 |
| *OsPRR3* | OsPRR3a | 13103.m02097 | LOC_Os03g17570.1 | 9768690 - 9759479 | 768 |
|  | OsPRR3b | 13103.m02098 | LOC_Os03g17570.2 | 9768690 - 9759479 | 768 |
|  | OsPRR3c | 13103.m02099 | LOC_Os03g17570.3 | 9768656 - 9759479 | 768 |
| *OsPRR4* | OsPRR4a | 13107.m05323 | LOC_Os07g49460.1 | 29616705 - 29629223 | 743 |
|  | OsPRR4b | 13107.m05320 | LOC_Os07g49460.2 | 29616732 - 29629223 | 743 |
|  | OsPRR4c | 13107.m05321 | LOC_Os07g49460.3 | 29616705 - 29629220 | 743 |
|  | OsPRR4d | 13107.m05322 | LOC_Os07g49460.4 | 29616705 - 29629220 | 743 |
| *OsPRR5* | OsPRR5a | 13111.m00578 | LOC_Os11g05930.1 | 2789002 - 2793735 | 700 |
|  | OsPRR5b | 13111.m00580 | LOC_Os11g05930.2 | 2788996 - 2793735 | 624 |
|  | OsPRR5c | 13111.m00581 | LOC_Os11g05930.3 | 2789002 - 2793735 | 624 |
|  | OsPRR5d | 13111.m00582 | LOC_Os11g05930.4 | 2789002 - 2793735 | 621 |
|  | OsPRR5e | 13111.m00579 | LOC_Os11g05930.5 | 2789002 - 2793729 | 601 |
| **SDRR** | | | | | |
| *OsETR2* | OsETR2a | 13104.m00795 | LOC_Os04g08740.1 | 4738375 - 4742348 | 764 |
|  | OsETR2b | 13104.m00796 | LOC_Os04g08740.2 | 4738885 - 4742348 | 764 |
| *OsETR4* | OsETR4 | 13107.m01627 | LOC_Os07g15540.1 | 9008499 - 9005169 | 778 |
